# Psychological Adjustment to Metastatic Cancer: A Comparative Longitudinal Analysis of Breast vs. Lung Cancer Patients

**DOI:** 10.3390/healthcare14081112

**Published:** 2026-04-21

**Authors:** Mitar Saveljic, Milovan Roganovic, Emir Muzurovic, Nikica Sutalo, Marin Mamic, Sanja Plestina

**Affiliations:** 1 Clinical Center of Montenegro, 81000 Podgorica, Montenegro; mil.roganovic@gmail.com (M.R.); emir.muzurovic@kccg.me (E.M.); 2 Faculty of Medicine, University of Montenegro, 81000 Podgorica, Montenegro; 3 Department of Surgery, University Hospital of Mostar, 88000 Mostar, Bosnia and Herzegovina; nikica.sutalo@tel.net.ba; 4 School of Medicine, University of Mostar, 88000 Mostar, Bosnia and Herzegovina; 5 Faculty of Health Studies, University of Mostar, 88000 Mostar, Bosnia and Herzegovina; 6 General County Hospital Požega, 34000 Požega, Croatia; mmamic@fdmz.hr; 7 Faculty of Dental Medicine and Health Osijek, Josip Juraj Strossmayer University of Osijek, 31000 Osijek, Croatia; 8 Department for Respiratory Diseases, University Hospital Centre Zagreb, 10000 Zagreb, Croatia; sanja.plestina@gmail.com; 9 School of Medicine, University of Rijeka, 51000 Rijeka, Croatia

**Keywords:** metastatic cancer, psychological distress, oncological treatment, faith and coping

## Abstract

**Background:** Psychological distress, including anxiety, stress, and depression, is common and expected in women with newly diagnosed metastatic breast (MBC) or lung (MLC) cancer. This study examined short-term changes in depression, anxiety, and stress and the associations of such changes with demographic, lifestyle, and religiosity factors. **Methods:** This prospective longitudinal study included 121 women (66 with MBC and 55 with MLC) who attended two oncology clinics in Montenegro between July 2024 and February 2025. Psychological symptoms were assessed at baseline (T1) and three months after treatment initiation (T2) using the Depression Anxiety Stress Scale-21 (DASS-21) and the Anxiety Sensitivity Index (ASI). Due to the non-normal distribution of residuals, multivariable quantile regression models (median regression, tau = 0.5) were used to examine independent predictors of psychological outcomes at T2, adjusting for baseline symptom severity, demographics, and clinical factors. **Results:** Baseline DASS-21 and ASI scores were comparable between the MBC and MLC patients. At three-month follow-up, depressive symptoms decreased in the overall cohort, driven by improvement among patients with MLC. Patients with MBC showed increased anxiety and stress over time, whereas patients with MLC showed reductions in depression and stress. At follow-up, anxiety was significantly higher in the MBC group than in the MLC group. The perceived importance of faith showed weak negative correlations with distress (ρ ranging from −0.227 to −0.242; all *p* = 0.01), while younger age was strongly associated with higher baseline distress in the MLC group (ρ = −0.431 to −0.688; all *p* < 0.001). In the multivariable median regression models, baseline symptom severity was the strongest predictor of psychological outcomes at T2 (*p* < 0.001). Additionally, cancer type was a significant independent predictor of depression at follow-up (B = −1.258; *p* < 0.001), with MLC associated with lower scores compared to MBC. **Conclusions:** Psychological distress is a common phenomenon during the first months after the diagnosis of metastatic cancer. Our results support the importance of early psychological assessment and tailored support in routine oncology practice.

## 1. Introduction

Cancer represents one of the most critical global public health burdens of modern medicine [1]. The complex etiology, insufficiently clear pathophysiology, and different clinical presentations, as well as the impact on patients’ quality of life, have made malignant diseases the central focus of modern medical research. GLOBOCAN data [2] show that lung cancer is the leading malignant disease in terms of both incidence and mortality worldwide, while breast cancer ranks second in incidence and fourth in mortality. Additionally, breast and lung cancers are the leading causes of morbidity and mortality among female patients. Although Montenegro lacks updated statistical data on this topic, preliminary data show that the cancer incidence and mortality rates in Montenegro align with global trends.

Despite extensive efforts in primary prevention and early detection (screening programs), patients are still frequently diagnosed with breast and lung cancer at the metastatic stage in clinical practice [3]. Numerous studies have shown that patients with malignant diseases, including women diagnosed with metastatic breast (MBC) and lung (MLC) cancer, exhibit high levels of anxiety and depression, fear of disease progression, fear of recurrence, and other psychological and psychiatric comorbidities [4,5,6,7]. These psychological comorbidities are frequently underdiagnosed [8] and can profoundly contribute to suboptimal treatment outcomes in patients with metastatic lung and breast cancer. This issue becomes even more important considering that previous research had indicated that the presence of depression and anxiety is a negative predictor of overall survival and occurrence of breast cancer recurrence [9].

The prevalence of anxiety and depression is notably high in patients with MBC. A study conducted in China [10] reported anxiety in 60.2% of patients and depression in 52.3%. Another study [11] reported a high prevalence of 36.9% for mild to severe depression among patients with MBC, while the prevalence of mild to severe anxiety was even higher at 44.2%. Similar results were reported in other previous studies [12].

As with MBC, most studies on the prevalence of anxiety and depression in patients with MLC have been conducted in Asian countries. According to data from China [13], the prevalence of depression and anxiety among MLC patients was 58.1% and 34.2%, respectively. Factors identified as contributing to higher levels of anxiety and depression included ECOG (Eastern Cooperative Oncology Group) performance status 3–4, administration of palliative treatment, prolonged hospitalization, comorbidities (e.g., arterial hypertension), and high TNM (Tumor, Node, Metastasis) stage [14,15].

The standard therapeutic approach for patients with MLC and MBC includes the combined use of hormonal therapy, immunotherapy, chemotherapy, and/or targeted therapy [16,17]. Symptomatic and supportive care—which includes psychotherapy—is considered standard in all modern oncology centers. This comprehensive therapeutic approach not only contributes to longer survival and higher survival rates but also significantly improves QoL.

Even though it is known that the diagnosis of an advanced malignant disease is associated with psychological distress, there is a lack of studies on how anxiety, depression, and stress change in the first stages after the diagnosis is made and during the first months of treatment. In particular, there is a lack of comparative studies across different metastatic cancer types. This gap is clinically relevant, as the first months after diagnosis represent a period marked by major emotional adjustment, prognostic uncertainty, and adaptation to treatment. A better understanding of how psychological symptoms change during this period and which psychological factors may be associated with treatment-related adaptation and clinical burden would contribute to the early identification of patients at higher risk of poor outcome [18,19]. Such information would be helpful to clinicians planning for supportive psychological interventions in routine oncology practice. Bearing in mind that the treatment of metastatic diseases is characterized by prognostic uncertainty and the need to frequently change the therapeutic protocol, elevated anxiety and depression in these patients may reflect reactive psychological distress during a period of prognostic uncertainty and treatment adaptation. A comparative assessment of women with MBC and MLC can therefore provide insight into whether the psychological trajectories differ between these two groups, whose contexts are characterized by distinct clinical and psychosocial factors.

Psychological distress in patients with metastatic cancer is shaped not only by disease burden and treatment-related factors but also by broader psychosocial and behavioral determinants [20]. Variables such as age, smoking status, and the perceived importance of faith may influence emotional adjustment through different pathways, including coping style, perceived control, health-related vulnerability, and access to psychological or social resources. This perspective is consistent with theoretical models of stress coping and adaptation, where psychological adjustment to a severe illness is understood as the result of interactions between disease burden, individual coping resources, and the broader psychosocial context. In this context, faith may function as a coping resource [21], whereas smoking status may reflect a broader behavioral and clinical vulnerability, particularly among patients with lung cancer.

In Montenegro, which has about 620,000 inhabitants, the treatment of oncology patients is centralized, and the availability of psychological support is limited due to the lack of staff and structured protocols. This results in an insufficient recognition of psychological symptoms and therefore inadequate treatment. Thus far, there is no study conducted on this topic in Montenegro. Addressing this gap is important for informing clinical pathways, triage for supportive services, and the development of context-appropriate psycho-oncological interventions.

The aim of this study was to assess changes in the levels of depression, anxiety, and stress among patients with MBC and MLC during the initial phase of treatment.

## 2. Methods

### 2.1. Study Design and Setting

Female patients newly diagnosed with MBC or MLC were recruited to take part in this prospective cohort study. The prospective observational design was chosen to evaluate variations in psychological outcomes after the metastatic cancer diagnosis and initiation of routine oncological treatment. The patients were prescribed systemic chemotherapy, in accordance with the standard clinical practice for MBC or MLC. Because this study was a real-world observational study, treatment approaches were heterogeneous and regimen-level data were not collected in a format suitable for stratified or modality-specific analyses. Accordingly, treatment type was not included as a covariate in the multivariable regression models.

This study was conducted at the Institute of Oncology and Radiotherapy, Clinical Center of Montenegro (MBC patients), and the Special Hospital for Pulmonary Diseases “Dr Jovan Bulajić” (MLC patients) during the period from 1 July 2024 to 1 February 2025. Ethical approval was obtained from the Ethics Committee of the Clinical Center of Montenegro (No. 03/01-32844/1). All participants provided written informed consent.

### 2.2. Sample Size Calculation

An a priori sample size calculation was performed using G*Power software (version 3.1). Based on paired comparisons of continuous outcomes between baseline and follow-up in the overall study population, assuming a medium effect size (Cohen’s dz = 0.5), a two-sided significance level of 0.05, and statistical power of 0.90, the minimum required sample size was estimated to be 44 participants. This calculation was performed for the primary longitudinal within-sample comparisons and was not specifically based on the requirements of the multivariable quantile regression analyses, which should therefore be interpreted as exploratory and with appropriate caution.

### 2.3. Participant Selection: Inclusion/Exclusion Criteria

The inclusion criteria were as follows: female sex; age ≥ 18 years; histopathologically confirmed diagnosis of MBC or MLC; Eastern Cooperative Oncology Group (ECOG) performance status ≤3; and ability to provide informed consent and complete questionnaires independently. The exclusion criteria included pre-existing psychiatric disorders diagnosed prior to the cancer diagnosis, current psychiatric treatment (including psychotherapy or counseling) or use of psychotropic medication, and cognitive impairment.

### 2.4. Recruitment and Study Procedures

Participants were recruited at both centers using identical eligibility criteria and assessment procedures. All eligible patients attending the participating institutions during the study period were approached consecutively. After eligibility screening and provision of written informed consent, participants completed baseline assessments prior to chemotherapy administration (T1). A follow-up assessment was conducted three months later (T2) during a routine clinical visit using the same procedures. The 3-month follow-up period was selected to capture the early phase of psychological adjustment after diagnosis and treatment initiation—a period characterized by emotional adaptation, prognostic uncertainty, and initial treatment-related experiences.

### 2.5. Data Collection and Assessment Setting

Questionnaires were completed in person and during regular visits. Prior to data collection, participants received standardized instructions emphasizing that the questionnaire items referred to emotional and psychological experiences rather than physical symptoms related to cancer or its treatment. No structured psychotherapy, mental health counseling, or self-help programs were routinely provided within the oncology settings during the study period. No participants were lost to follow-up between baseline and the three-month assessment.

### 2.6. Instruments

Demographic and clinical data (age, smoking status, education level, comorbidities, and perceived importance of faith in life) were collected using a structured questionnaire. The perceived importance of faith in life was assessed using a single self-reported item and was included as an exploratory psychosocial variable rather than a comprehensive measure of religiosity or spirituality. Psychological outcomes were assessed using the following two self-report instruments:

(1) Depression Anxiety Stress Scale–21 (DASS-21), comprising three 7-item subscales assessing depression, anxiety, and stress, was used to evaluate psychological distress. A previous Serbian validation study [22] demonstrated strong internal consistency (Cronbach’s α = 0.87 for the depression scale, 0.85 for the anxiety scale, 0.89 for the stress scale, and 0.93 for the total scale). Higher scores on the DASS-21 subscales suggest progressive escalation in the intensity of symptoms related to depression, anxiety, and stress.
(2) Anxiety sensitivity was assessed using a validated Croatian version of the 16-item Anxiety Sensitivity Index (ASI) [23], which showed good internal consistency (Cronbach’s α = 0.86 for the total ASI score).

The DASS-21 and Anxiety Sensitivity Index (ASI) have demonstrated good reliability and validity in their original versions. In the present study, internal consistency was evaluated using Cronbach’s alpha coefficients and is reported in the Results section. Assessments were conducted at baseline (prior to treatment initiation) and after three months of treatment.

### 2.7. Statistical Analyses

Categorical variables were summarized as counts and percentages and compared using the chi-squared test. The internal consistency of the measurement instruments was assessed using Cronbach’s alpha. Normality of continuous variables was evaluated with the Shapiro–Wilk test. Continuous variables are presented as medians with interquartile ranges (IQRs). Between-group comparisons of continuous variables by cancer type were performed using the Mann–Whitney U test, while within-group comparisons between baseline and follow-up were conducted using the Wilcoxon signed-rank test. Hodges–Lehmann median differences with 95% confidence intervals were reported for nonparametric comparisons. Associations between continuous variables were assessed using Spearman’s rank correlation coefficient (ρ).

To determine the predictors of depression, anxiety, and stress at the second measurement point (T2), multivariable quantile regression (median regression, tau = 0.5) was performed. This robust approach was chosen because the Shapiro–Wilk test and visual inspection of residual distributions indicated significant deviations from normality (*p* < 0.05), accompanied by mild skewness and the presence of several influential observations (outliers) identified through standardized residuals and leverage diagnostics. Given that ordinary least squares (OLS) regression relies on assumptions of normally distributed and homoscedastic residuals, violations of these assumptions may bias standard errors and affect statistical inference. Quantile regression, which estimates conditional medians rather than means, is less sensitive to outliers and distributional irregularities and therefore provides a more robust estimation of central tendency under such conditions. The validity of the model was confirmed by checking for multicollinearity, which was ruled out by the low VIF values obtained (below 2.1). The explanatory power of the models was expressed through the Pseudo “R2” index, which represents the relative reduction in absolute deviations.

All statistical tests were two-sided, with statistical significance set at α = 0.05. Analyses were performed using MedCalc^®^ Statistical Software version 23.3.7 (MedCalc Software Ltd., Ostend, Belgium; 2025).

## 3. Results

A total of 121 female patients were included in the study, of whom 66 were diagnosed with MBC (54.5%) and 55 with MLC (45.5%). Patients with MLC were significantly older than those with MBC (Hodges–Lehmann median age difference = 12 years; 95% CI: 8 to 16; Mann–Whitney U test, *p* < 0.001) and were more frequently current smokers (93% vs. 33%, chi-squared test, *p* < 0.001). Significant differences between groups were also observed in terms of educational level and self-reported importance of faith in life (chi-squared test, *p* = 0.01 and *p* < 0.001, respectively). However, when perceived importance of faith was analyzed as an ordinal score (median rating), no significant difference between groups was observed. The prevalence of major comorbidities did not differ significantly between groups (Table 1).

As previously mentioned, depression, anxiety, and stress were measured using the DASS-21 at the time of cancer diagnosis (T1) and after three months, i.e., after three cycles of chemotherapy (T2). This instrument demonstrated high internal consistency at both assessments (Cronbach’s α = 0.936 at T1 and 0.927 at T2). Scores on each subscale range from 0 to 21, with higher scores indicating greater symptom severity. Anxiety sensitivity was assessed using the ASI, which also showed high internal consistency at both time points (Cronbach’s α = 0.917 at T1 and 0.891 at T2).

At baseline, no statistically significant differences between groups were observed across the DASS-21 domains. At follow-up, anxiety scores were significantly higher in patients with MBC compared with those with MLC, while differences in depression and stress were not statistically significant. No statistically significant between-group differences in ASI scores were observed at either baseline or follow-up (Table 2).

In the overall sample, depressive symptoms decreased significantly over time (median difference = 0; 95% CI: −1 to 0). Among patients with MBC, anxiety and stress increased from baseline to follow-up (median differences = 0.5 [95% CI 0 to 1] and 0.5 [95% CI 0 to 1], respectively). In contrast, patients with MLC showed reduction in depression (median difference = −2; 95% CI: −2.5 to −0.5) and stress (median difference = −1.5; 95% CI: −2.5 to −0.5). No significant changes were observed for anxiety and ASI scores in the overall sample, nor for depression and ASI scores among patients with MBC, or anxiety and ASI scores among patients with MLC (Table 3).

The correlations between age, perceived importance of faith in life, and DASS-21 and ASI scores at baseline (T1) and follow-up (T2) are shown in Table 4. In the overall sample, greater importance of faith was associated with lower DASS-21 depression, anxiety, and stress scores at both time points, although some of these correlations were weak despite reaching statistical significance (|Rho| < 0.40). Among patients with MLC, younger age showed strong negative correlations with baseline DASS-21 depression, anxiety, and stress scores (ρ up to −0.69), but the associations were weakened at follow-up. In contrast, correlations between age and DASS-21 scores were generally weak and not statistically significant in patients with MBC. No consistent or strong correlations were observed between ASI scores and age or importance of faith in life across groups or time points (Table 4).

To examine predictors of psychological distress at the second measurement point (T2), three separate multivariate quantile regressions on the median were conducted. This robust approach was chosen because the residuals significantly deviated from a normal distribution (*p* < 0.001) and it could ensure that the models were robust to outliers in the sample. The scores on the depression, anxiety, and stress subscales (DASS-21) at T2 were used as dependent variables, while the predictor set consisted of baseline symptom scores (T1), demographic characteristics (age), clinical variables (cancer type: 1—breast, 2—lung; smoking status), and level of religiosity (importance of faith in life). The depression model was statistically significant and explained 50.2% of the variability around the median (Pseudo R^2^ = 0.502). The strongest significant predictor was baseline depression (B = 0.839; *p* < 0.001), with higher scores at T1 predicting significantly higher scores at T2. After controlling for baseline symptom scores and other variables, cancer type was the only remaining significant independent predictor (B = −1.258; *p* < 0.001). The negative direction of the coefficient indicated that patients with lung cancer had significantly lower median scores on the depression subscale compared to patients with breast cancer. In the depression model, the effect of age was of borderline statistical significance (B = 0.032; *p* = 0.058). In the anxiety model, which explained 61.4% of the variability around the median (Pseudo R^2^ = 0.614), the only statistically significant predictor was baseline anxiety (B = 0.862; *p* < 0.001), indicating the high stability of anxiety symptoms over time. Similar findings were obtained for the stress model (Pseudo R^2^ = 0.421), in which only initial stress made a significant contribution (B = 0.714; *p* < 0.001) (Table 5).

## 4. Discussion

With respect to the demographic characteristics of the participants, patients in the MLC group were, on average, older, and—as expected—had a higher proportion of smokers. When analyzing the DASS-21 results in the first three months of receiving treatment, depressive symptoms decreased in the overall sample, driven primarily by a decline among patients with MLC, whereas no meaningful change was observed among patients with MBC. Previous studies [17,24] suggest that surgical treatment (mastectomy) contributes to a reduction in depression levels; however, most of these studies have been conducted in patients with non-metastatic breast cancer. The importance of reducing depressive symptoms to prolong survival in MBC patients has been clearly demonstrated in previous studies [25]. However, the available literature mainly discusses the effects of physical and complementary therapies on alleviating depressive symptomatology in cancer patients [26,27], and there is a lack of evidence on how oncological therapeutic protocols affect depressive symptomatology.

In this prospective observational study, patterns of change in depression, anxiety, and stress during the first three months after diagnosis and treatment initiation differed by cancer type. Although several within-group changes reached statistical significance, the observed effect sizes were generally small to moderate, suggesting that some changes may have limited clinical relevance at the individual level. These findings should therefore be interpreted cautiously and considered primarily as descriptive of symptom dynamics during the early treatment phase.

In contrast to the findings related to depression, anxiety trajectories differed between the two groups. Patients with MBC showed a significant increase in anxiety at follow-up, whereas no significant change was observed among patients with MLC. As noted for depression in the context of MBC, there is evidence suggesting that anxiety levels may influence treatment outcomes in patients with MBC [28]. Some studies [14,29,30] have reported lower anxiety levels among patients receiving immunotherapy; however, because our patient cohort received multiple treatment modalities and we could not stratify by regimen, we could not attribute observed changes to a specific treatment type. Differences between MLC and MBC may also be attributed to different treatment-related symptom profiles and biological pathways; for example, systemic inflammation, fatigue, sleep disruption, and endocrine symptoms may differentially shape anxiety and mood during early therapy, potentially contributing to divergent psychological trajectories.

Another possible explanation for the differing anxiety trajectories observed in women with MBC and MLC may lie in the distinct psychosocial and clinical contexts of these diseases. In patients with MBC, anxiety may be reinforced by concerns related to body image, femininity, and the prolonged emotional burden associated with a metastatic diagnosis, even during active treatment [31,32]. In contrast, psychological responses in patients with MLC may be shaped more strongly by physical symptom burden, comorbidity profile, and adaptation to a clinical presentation that is often more overtly severe. Differences in illness perception, awareness of prognosis, and treatment-related expectations may also contribute to the distinct patterns observed between the two groups. These interpretations remain speculative, as such factors were not directly measured in the present study, but they may help contextualize the observed differences in anxiety trajectories during the early phase of metastatic disease.

Stress trajectories also differed by cancer type. Stress levels increased significantly in patients with MBC, whereas patients with MLC showed a significant reduction over the three-month follow-up period. The importance of stress reduction lies not only in improving patients’ QoL but also in the fact that elevated stress levels may negatively affect the effectiveness of certain drugs used to treat patients with metastatic malignancies [33].

Baseline-adjusted multivariable quantile regression demonstrated that while baseline symptom status was the strongest predictor, cancer type remained a significant independent predictor of depression at follow-up (B = −1.258; *p* = 0.0), whereas it did not reach statistical significance for the prediction of anxiety and stress at follow-up.

The correlations observed at baseline, particularly in patients with MLC, became weaker at follow-up and, in several cases, were small in magnitude, suggesting limited practical significance and possible regression to the mean. Unmeasured psychosocial factors such as social support, financial strain, caregiving responsibilities, and access to psycho-oncological services may also differ between groups and could have contributed to the observed divergence in early symptom patterns, particularly the absence of improvement among MBC patients.

In our cohort, the influence of age was most evident among MLC patients at baseline. Although the association of age with psychological distress weakened over time, the borderline significance of age in our robust regression model (B = 0.032; *p* = 0.058) suggests it remains a relevant factor in identifying patients at risk for persistent distress. Similar findings have been reported in other studies [34,35], indicating that younger patients tend to exhibit higher levels of distress and concern.

Faith has been recognized as an important factor contributing to the psychological resilience of patients undergoing treatment for malignant diseases. Its impact is particularly pronounced in those with metastatic disease. For these patients, faith can serve as a key coping mechanism, providing them with a sense of hope and psychological stability [36,37]. Statistically significant negative correlations between levels of anxiety, depression, and stress and the perceived importance of faith in life were also demonstrated in our cohort. In the present study, faith was assessed using a single self-reported item, which does not capture the multidimensional nature of religiosity or spiritual coping. Therefore, this variable should be understood as an exploratory psychosocial indicator rather than a comprehensive measure of religiosity.

In our study, the dominant predictor of psychological outcomes in the first months of treatment was the severity of initial symptoms: patients who entered oncology treatment with a higher level of depression, anxiety, or stress also reported a higher level of these symptoms after three months, which points to a pronounced continuity of distress during the early phase of treatment. These findings suggest the relative stability of distress over time rather than clearly identifiable mechanisms of change. It is important to emphasize that patients with MLC showed a lower level of depression compared to patients with MBC. Taken together, these results, as well as other previously published findings [38,39], underline the clinical importance of early identification of psychological distress at the start of oncological treatment.

The observed differences between patients with MBC and those with MLC may also have been influenced by treatment-related factors, since patients were managed with different systemic modalities whose side-effect profiles may differ substantially.

The most significant strength of this study is its prospective design, allowing for the monitoring of psychological changes following the initiation of oncological treatment. Additionally, the simultaneous comparison of two distinct patient groups (MBC and MLC) offers valuable insights into disease-specific emotional responses and treatment effects.

It is important to acknowledge a number of essential limitations associated with the research design. First, while the sample size is sufficient for initial analysis, it restricts the generalizability of the results. In addition, the single-country setting and the inclusion of only female patients also limit the external validity and generalizability of the findings. Second, it is hard to link the observed symptom changes to treatment since there is no control group that did not receive standard oncological care. Regression to the mean, natural psychological adaptation following diagnosis, and other time-related factors not related to treatment might all have contributed to the changes in psychological symptoms. Third, assessments were conducted at only two time points, which made it impossible to model long-term symptom trajectories and limited the ability to differentiate between measurement error and actual intra-individual change. The exploratory nature of subgroup analyses also highlighted the possibility of Type I error. Additionally, despite being widely used and validated, self-report measures like the DASS-21 and ASI may be prone to response biases such as recall bias and social desirability bias.

This study did not fully address the factors that can influence psychological symptoms in patients with advanced cancer. Symptoms of depression and anxiety can be exacerbated by the physical burden of the disease, such as pain, fatigue, or side effects of treatment, and be alleviated by social and environmental support. Detailed evaluations of physical symptoms, social support, and specific treatment regimens were not carried out, and the effects of confounding factors could not be ruled out, even though major comorbidities were included and treatment was given in standardized clinical settings. In addition, the perceived importance of faith was assessed using a single item, which limits the ability to draw broader conclusions about religiosity as a multidimensional construct. Future studies should incorporate more comprehensive and multidimensional measures of religiosity or spiritual coping. Finally, there are restrictions associated with the use of self-reporting methods. More thorough psychological profiling and larger, more diverse samples are required in future research to completely understand the emotional strain faced by MBC and MLC patients and their mental health needs.

## 5. Conclusions

In conclusion, psychological distress trajectories during the first three months after diagnosis differ significantly between women with MBC and those with MLC. While reductions in depression and stress were observed in the MLC cohort, patients with MBC exhibited a more persistent or escalating pattern of anxiety and stress. Baseline symptom severity was the strongest predictor of distress at follow-up, suggesting that early psychological screening may help identify patients at increased risk of ongoing emotional burden. In resource-limited settings such as Montenegro, where dedicated psycho-oncological support is scarce, brief screening tools could be used as a pragmatic triage approach to prioritize patients with high baseline distress and those with MBC for closer follow-up, referral, or supportive intervention within routine oncology care.

## Figures and Tables

**Table 1:** Descriptive characteristics of the study participants.

Characteristics	MBC	MLC	All	*p* * Value
N (%)	66 (54.5)	55 (45.5)	121	
Age, median (IQR)	50 (45–63)	65 (58–68)	58 (45–68)	<0.001 ^†^
Comorbidities, N (%)				
Myocardial infarction	2 (3)	3 (6)	5 (4)	0.66
Diabetes	3 (5)	2 (4)	5 (4)	>0.99
Educational level, N (%)				
No primary education	3 (5)	0	3 (2)	0.01
Primary education	8 (12)	4 (7)	12 (10)	
Secondary education	40 (61)	48 (87)	88 (73)	
Post-secondary education	6 (9)	2 (4)	8 (7)	
Bachelor’s degree or higher	9 (14)	1 (2)	10 (8)	
Smoking status, N (%)				
Current smoker	22 (33)	51 (93)	73 (60)	<0.001
Past smoker	10 (15)	4 (7)	14 (12)	
Non-smoker	34 (52)	0	34 (28)	
Perceived importance of faith in life, N (%)				
Not at all	2 (3)	1 (2)	3 (3)	<0.001
A little	5 (8)	1 (2)	6 (5)	
Moderately	16 (25)	22 (40)	38 (32)	
Quite a bit	16 (25)	21 (38)	37 (31)	
Very much	26 (40)	10 (18)	36 (30)	
The importance of faith in life, median (IQR)	4 (3–5)	4 (3–4)	4 (3–5)	0.12

* Chi-squared test; ^†^ Mann–Whitney U test. Bold values indicate statistically significant differences (*p* < 0.05). Abbreviations: IQR—interquartile range; MBC—metastatic breast cancer; MLC—metastatic lung cancer.

**Table 2:** Changes in DASS-21 depression, anxiety, and stress scores and ASI scores over time by cancer type.

	Median (IQR)		Difference ^†^	95% CI	*p* * Value
	MBC	MLC			
Baseline (T1)					
Depression	5 (1–11)	8 (5–12)	2	0 to 4	0.06
Anxiety	6 (2–13)	5 (3–9)	−1	−3 to 1	0.25
Stress	8 (4–14)	9 (6–12)	0	−2 to 2	0.94
ASI	36 (28–50)	36 (31–48)	0	−5 to 4	0.96
After 3 months of treatment (T2)					
Depression	5.5 (3–12)	6 (4–9)	0	−2 to 2	0.98
Anxiety	8 (3–13)	5 (2–8)	−2	−4 to 0	0.01
Stress	8 (5–15)	7 (5–10)	−2	−3 to 0	0.09
ASI	38 (29–49)	37 (33–43)	−1	−6 to 3	0.60

* Mann–Whitney U test; ^†^ Hodges–Lehman median difference; bold values indicate statistically significant differences (*p* < 0.05). Abbreviations: IQR—interquartile range; MBC—metastatic breast cancer; MLC—metastatic lung cancer; CI—confidence interval; ASI—Anxiety Sensitivity Index.

**Table 3:** Changes in DASS-21 depression, anxiety, stress, and ASI scores over time by cancer type.

	Median (IQR)		Difference	95% CI	*p* * Value
	T1	T2			
All patients					
Depression	7 (3–12)	6 (3–10)	0	−1 to 0	**0.03**
Anxiety	6 (2–10)	6 (3–10)	0.5	0 to 0.5	0.15
Stress	9 (5–13)	8 (5–11)	0	−0.5 to 0	0.27
ASI	36 (30–48)	37 (32–46)	0.5	0 to 1	0.16
MBC					
Depression	5 (1–11)	5.5 (3–12)	0	0 to 0.5	0.25
Anxiety	6 (2–13)	8 (3–13)	0.5	0 to 1	0.005
Stress	8 (4–14)	9 (5–15)	0.5	0 to 1	0.04
ASI	36 (28–50)	38 (29–49)	0.5	0 to 1	0.15
MLC					
Depression	8 (5–12)	6 (4–9)	−2	−2.5 to −0.5	0.002
Anxiety	5 (3–9)	5 (2–8)	0	−1 to 0.5	0.62
Stress	9 (6–12)	7 (5–10)	−1.5	−2.5 to −0.5	0.01
ASI	36 (31–45)	37 (33–43)	0.5	−1 to 2.5	0.49

* Wilcoxon test (Hodges–Lehmann median difference). Bold values indicate statistically significant differences (*p* < 0.05). Abbreviations: IQR—interquartile range; T1—baseline; T2—after 3 months of treatment; MBC—metastatic breast cancer; MLC—metastatic lung cancer; CI—confidence interval; ASI—Anxiety Sensitivity Index.

**Table 4:** Correlations between age, importance of faith in life, and DASS-21 and ASI scores at baseline and follow-up.

	Spearman’s Correlation Coefficient Rho (ρ) (*p* Value)			
	Baseline (T1)		After 3 Months of Treatment (T2)	
	Age	Perceived Importance of Faith in Life	Age	Perceived Importance of Faith in Life
All patients				
Depression	0.101 (0.27)	**−0.235 (0.01)**	0.091 (0.32)	**−0.242 (0.01)**
Anxiety	−0.006 (0.95)	**−0.233 (0.01)**	−0.031 (0.74)	−0.159 (0.08)
Stress	−0.002 (0.98)	−0.134 (0.14)	−0.005 (0.96)	**−0.227 (0.01)**
ASI	0.036 (0.69)	−0.138 (0.13)	0.068 (0.46)	−0.075 (0.42)
MBC				
Depression	0.209 (0.09)	−0.230 (0.07)	0.217 (0.08)	−0.218 (0.08)
Anxiety	0.164 (0.19)	−0.234 (0.06)	0.133 (0.29)	−0.196 (0.12)
Stress	0.201 (0.10)	−0.189 (0.13)	0.181 (0.15)	**−0.272 (0.03)**
ASI	0.204 (0.10)	−0.190 (0.13)	0.219 (0.08)	−0.203 (0.10)
MLC				
Depression	**−0.653 (<0.001)**	−0.129 (0.35)	**−0.319 (0.02)**	−0.271 (0.05)
Anxiety	**−0.431 (<0.001)**	**−0.313 (0.02)**	−0.131 (0.34)	−0.207 (0.13)
Stress	**−0.688 (<0.001)**	−0.068 (0.62)	−0.259 (0.06)	−0.192 (0.16)
ASI	**−0.449 (0.001)**	−0.066 (0.64)	−0.253 (0.06)	0.129 (0.35)

Bold values indicate statistically significant correlations (*p* < 0.05). Abbreviations: MBC—metastatic breast cancer; MLC—metastatic lung cancer; ASI—Anxiety Sensitivity Index.

**Table 5:** Multivariable linear and quantile regression models of psychological outcomes at T2.

Outcomes and Predictors	B	95% CI (Lower; Upper)	*p*
DEPRESSION (T2)			
(Constant)	1.081	(−1.682; 4.592)	0.361
Baseline score (T1)	0.839	(0.751; 0.946)	<0.001
Cancer type	−1.258	(−1.907; −0.663)	0.000
Age	0.032	(−0.001; 0.060)	0.058
Perceived importance of faith in life	−0.161	(−0.335; 0.282)	0.865
Smoking status	0.145	(−0.362; 0.345)	0.985
Model Fit		Pseudo R^2^ = 0.502	
ANXIETY (T2)			
(Constant)	0.322	(−2.114; 2.758)	0.794
Baseline score (T1)	0.862	(0.784; 0.940)	<0.001
Cancer type	−0.942	(−2.015; 0.131)	0.086
Age	0.018	(−0.012; 0.048)	0.239
Perceived importance of faith in life	0.114	(−0.224; 0.452)	0.508
Smoking status	0.231	(−0.412; 0.874)	0.479
Model Fit		Pseudo R^2^ = 0.614	
STRESS (T2)			
(Constant)	2.145	(−1.556; 5.846)	0.254
Baseline score (T1)	0.714	(0.602; 0.826)	<0.001
Cancer type	−0.642	(−1.894; 0.610)	0.312
Age	0.008	(−0.024; 0.040)	0.621
Perceived importance of faith in life	−0.214	(−0.584; 0.156)	0.255
Smoking status	0.312	(−0.521; 1.145)	0.460
Model Fit		Pseudo R^2^ = 0.421	

Note: T1—baseline; T2—after 3 months of treatment; B—unstandardized quantile regression coefficient (τ = 0.5); CI—confidence interval; *p*—level of statistical significance; Pseudo R^2^—coefficient of determination for quantile regression.

## Data Availability

The data presented in this study are available from the corresponding author upon request.
